# AI-generated videos in medical science popularization and education: a scoping review

**DOI:** 10.3389/fpubh.2026.1921115

**Published:** 2026-08-18

**Authors:** Jiejia Ma, Xiujun Zheng, Lijin Wang, Jiachun Wu, Zimo Yang, Siyu Wei, Ruibin Huang

**Affiliations:** 1 Department of Radiology, The First Affiliated Hospital of Shantou University Medical College, Shantou, China; 2 Shantou University Medical College, Shantou, China; 3 Center for Faculty Development, The First Affiliated Hospital of Shantou University Medical College, Shantou, China

**Keywords:** AI generation procedure, evaluation tools, generative artificial intelligence, medical education, patient education, prompts, text to video

## Abstract

**Objectives:**

This scoping review aimed to systematically review research on the application of artificial intelligence-generated (AI-generated) videos for the general public education, patient education, medical student education, and physician education, and propose a framework covering the entire video production process.

**Methods:**

Arksey and O’Malley’s scoping review guidelines were followed, using the PRISMA-ScR checklist. Studies were identified from PubMed, Web of Science, China National Knowledge Infrastructure and Google Scholar using keywords and medical subject headings terms related to “AI-generated”, “evaluation” and “video”, and screened for relevance, with any duplicates removed.

**Results:**

The initial 1,206 articles were reduced to 10 for review. Of these, half failed to clearly document the prompts used. Those that did provided only brief descriptions based on the application context or keywords, and the AI-generated videos consistently received low scores on validated instruments such as the Global Quality Scale (GQS), with most scoring 2 out of 5, indicating limited suitability for patient education. Comprehensive quality assessments were rare: only one study used three distinct evaluation tools, and the rest used one or two or none at all, revealing significant shortcomings in the standardization and comprehensiveness of the overall evaluation framework.

**Conclusion:**

To the best of our knowledge, this is among the first scoping reviews to synthesize the emerging evidence on AI-generated videos for the general public education, patient education, medical student education, and physician education. Few studies are reported and they demonstrate considerable heterogeneity. We identified insufficient standardization in prompts, generation pathways, tools, and evaluation instruments. We propose a procedural framework that may guide and inform future research in this field.

## 1 Introduction

There has been a rapid rise in short-form video platforms in recent years. The use of these platforms for medical science popularization and the education of patients, healthcare professionals, and the public has expanded substantially, gradually reshaping traditional educational approaches (1). Previous studies have shown that educational videos can improve patient knowledge, self-efficacy, satisfaction, and engagement with healthcare, supporting their value as effective tools for health communication and education (2–4). For example, McIntyre et al. discovered that educational videos on atrial fibrillation, created by clinical physicians, significantly enhanced patients’ knowledge about the condition (3). Despite these advanxtages, the production of high-quality medical educational videos often requires substantial investments of time, expertise, and technical resources. For example, manual video development may require extensive multidisciplinary collaboration and considerable production costs (5, 6). Furthermore, a recent scoping review of manually produced patient educational videos reported considerable variability in both quality and educational effectiveness, highlighting ongoing challenges in achieving consistent and scalable educational outcomes (7). These limitations underscore the need for more efficient, cost-effective, and scalable approaches to medical video production, creating opportunities for the application of generative artificial intelligence technologies.

Advances in artificial intelligence (AI) have led to the widespread availability of new methods of generating videos, particularly those using “text-to-video” (T2V) techniques, and these have been widely adopted across various fields such as higher education (8). A substantial body of research has corroborated the technology’s efficacy and considerable application potential. In 2025, a study by Pellas evaluated AI-generated instructional videos in pre-service science teachers’ problem-based learning. It found significant improvements in participants’ task performance, knowledge retention, and self-efficacy across all evaluative measures (9). Luo et al. developed an AI-based system to generate personalized medical education videos for emergency department discharge instructions and found that these videos achieved higher ratings in completeness, accuracy, and accessibility than alternative methods of patient education (10). However, studies investigating the use of AI-generated videos for medical science popularization and the education of patients, healthcare professionals, and the public remain limited (11). Meanwhile, several studies identified concerns regarding the quality and reliability of AI-generated medical educational videos. These concerns could be broadly categorized into (1) factual medical inaccuracies, (2) inappropriate or misleading visual representations, (3) insufficient explanation of medical concepts, (4) incomplete risk communication, and (5) limitations in readability and audience appropriateness (12, 13). In this review, we use the term “AI-generated medical educational videos” as an overarching concept encompassing medical science popularization, public health education, patient education, and healthcare professional education. Although these contexts differ in target audiences, educational objectives, and evaluation outcomes, they share a common purpose of facilitating medical knowledge communication through AI-generated video content. Furthermore, despite growing interest in this area, the existing evidence has not yet been comprehensively synthesized within a scoping review framework. Therefore, this scoping review aimed to systematically map the current literature on AI-generated videos for the general public education, patient education, medical student education, and physician education, identify key methodological and research gaps, and propose a conceptual framework to inform future research and practice.

## 2 Methods

This review followed the framework proposed by Arksey and O’Malley (14), using five steps: (1) identifying the research question; (2) identifying relevant studies; (3) study selection; (4) charting the data, and (5) collating, summarizing and reporting the results. In parallel, we also followed the Preferred Reporting Items for Systematic Reviews and Meta-Analyses extension for Scoping Reviews (PRISMA-ScR) checklist (15). The completed checklist is provided in the Supplementary Material A. Ethical approval was not required for this scoping review because it relied exclusively on publicly available data from previously published studies. No primary data involving human participants or animals were collected or analyzed. A review protocol was not previously registered. Although the review protocol was not formally registered, the review team developed an internal protocol before commencing study selection. This protocol specified the review objectives, eligibility criteria, search strategy, study selection process, and data extraction items. A predefined data extraction form was created and piloted by the reviewers prior to full data extraction. Any disagreements during screening and extraction were resolved through discussion among the research team until consensus was reached.

### 2.1 Identification of the research question

Research on AI-generated videos for the general public education, patient education, medical student education, or physician education is fragmented, with no existing review summarizing the evidence. This study conducted a scoping review, using broad research questions to map evidence in this field, clarify the current research status, and identify knowledge gaps. The research questions covered were: (i) What is the current development status of AI-generated videos for the general public education, patient education, medical student education, and physician education? (ii) What are the characteristics of their prompt design, generation paths, tool selection, and the selection of video evaluation tools?

### 2.2 Identifying relevant studies

Two researchers (*** and ***) searched four electronic databases: PubMed, Web of Science, Google Scholar and China National Knowledge Infrastructure (CNKI). Google Scholar was included as a supplementary source to capture grey literature and studies that may not be indexed in the three traditional databases, which is particularly important given the emerging nature of this field. Initial keywords relevant to the research objectives were identified, such as ‘*artificial intelligence generated video*’, ‘*popular science education*’, and ‘*medicine*’. A preliminary search was carried out in the four electronic databases using these keywords. The search terms were then analyzed, discussed, and optimized to fit the specific requirements of each database. These new search terms were further reviewed and refined by a third researcher (***). The search terms corresponding to the different platforms are compiled in Supplementary Material B.

### 2.3 Eligibility criteria

Studies were selected based on specific inclusion and exclusion criteria. The inclusion criteria were as follows: (1) original research studies on AI-generated videos for the general public education, patient education, medical studen education, or physician education; (2) including randomized controlled trials, quasi-experimental research, observational research and mixed methods research; (3) published from 2017 to February 18, 2026, and (4) published in either English or Chinese. The exclusion criteria were as follows: (1) comments, editorials or reviews, and (2) publications, theses or unpublished papers with only an abstract, where complete data could not be obtained. To ensure comprehensive coverage, our search strategy and eligibility criteria did not impose any restrictions on the type of health education, provided that the study population fell into one of the four predefined groups: general public, patients, medical students, or physicians.

### 2.4 Study selection

**Figure 1:**
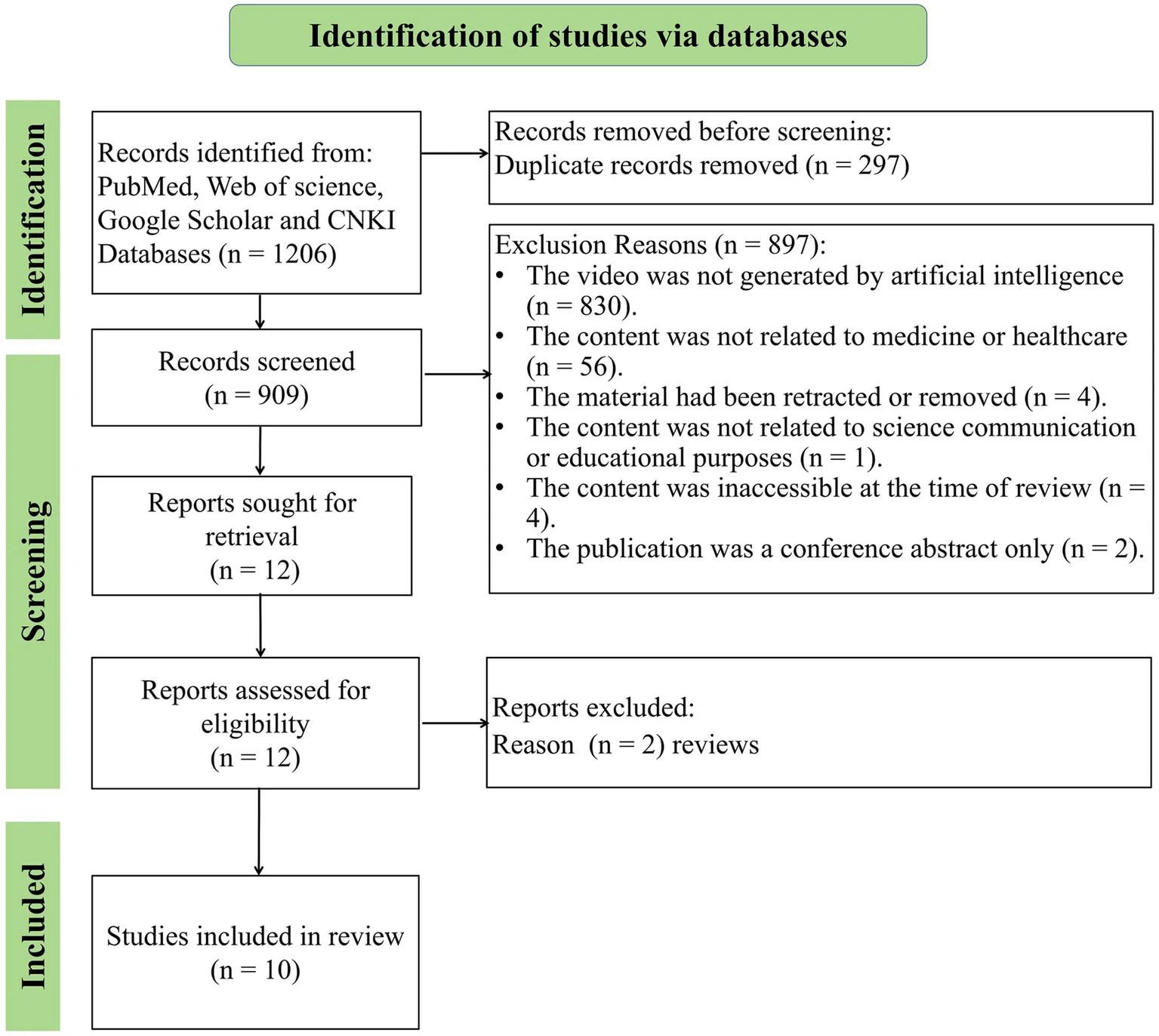
PRISMA flow diagram of article screening process.

On February 18, 2026, two independent researchers (*** and ***) conducted searches on four databases using the search terms from Supplementary Material B. The resulting literature was imported into EndNote for document management purposes. Following the removal of duplicates, the remaining articles were screened by title and abstract against the eligibility criteria, resulting in the exclusion of irrelevant studies. The two researchers then thoroughly read the remaining studies and conducted further screening. In accordance with the requirements of the PRISMA Extension Guidelines, no formal assessment of study quality was conducted. The reasons for exclusion were documented and reported (Figure 1).

### 2.5 Charting the data

Excel database software was used for the data charting and chart creation. Data charting was performed independently by one researcher (***), and cross-checked and validated by the second researcher (***) to ensure the accuracy and consistency of the data. The charted information included the first author, the country where the study was conducted, the year of publication, the research objectives, methodological characteristics, and conclusions. We also collected data about tools used to assess video quality and content, including Global Quality Score (GQS) (16), Modified DISCERN (mDISCERN) (17), and Journal of the American Medical Association (JAMA) benchmark criteria (18). We also included information about whether studies used self-developed scales as evaluation tools, and the use of the intraclass correlation coefficient (ICC). This is frequently used to quantify intra-rater reliability levels because of its scientific rigor and objectivity, enabling a precise assessment of the consistency of evaluations conducted by the same rater across different contexts (19). Any disagreements were resolved through discussion, with arbitration if needed by a third reviewer (***). The studies were categorized into three typologies based on research design: qualitative research, quantitative research, and mixed-methods research (integrating both qualitative and quantitative approaches).

Drawing on the included studies and existing AI video-generation workflows, we synthesized five pathways for AI-generated text-to-video (T2V) production. These pathways represent an author-developed analytical framework rather than an established taxonomy and are described as follows (20–22):

- Path I: Open-Source Commercial T2V, which is a direct T2V generation approach that adopts open-source models, supports local deployment, features strong privacy protection and low cost, and is suitable for privacy-sensitive medical scenarios.
- Path II: Closed-Source Professional T2V, which is a high-fidelity T2V generation method based on closed-source professional platforms, and provides realistic visual effects and professional camera motion for high-quality medical demonstration.
- Path III: Closed-Source Entry-Level T2V, which is a user-friendly T2V generation strategy using consumer-level closed-source tools, with excellent Chinese language adaptation and zero learning threshold, applicable for rapid medical science popularization.
- Path IV: Stepwise Generation, which is a structured generation pipeline that includes text-to-script conversion, shot segmentation, frame-by-frame video generation, and post-editing splicing, enabling precise control of complex medical content and sequential logic.
- Path V: Enhanced Generation, which is an integrated one-stop video production approach that combines AI-generated video generation, dubbing, background music matching, and subtitle addition, directly outputting publishable finished videos.

This study intentionally incorporated the general public, patients, medical students, and physicians into a single analytical framework, guided by three principal considerations. First, in real-world settings, AI-generated medical science popularization videos are inherently disseminated to all these groups, and a given video is often viewed simultaneously by individuals with widely differing knowledge backgrounds. Therefore, analyzing any single group in isolation would detach the evaluation from actual usage contexts. Second, the core metrics we employed are fundamentally applicable across all populations, despite their differences in prior knowledge and intended purposes. This general applicability provides a theoretical and methodological basis for meaningful unified comparisons. Third, the limited number of primary studies currently available in this emerging field (*n* = 10) precludes conducting fully stratified syntheses for each population without compromising statistical robustness and interpretability. A pooled analytical approach thus represents a pragmatic and necessary first step for mapping the current evidence landscape. While we acknowledge potential heterogeneity across populations, the limited data preclude formal stratification; we therefore note population-specific observations descriptively in our synthesis and address their implications in the Discussion.

### 2.6 Collating, summarizing and reporting the results

Qualitative content analysis (14) and thematic analysis (23) were used to analyze and synthesize the studies included in the review. The process included six interrelated stages: (1) Familiarization with the data through repeated reading of the full text; (2) Generating initial coding related to the AI tool selection, evaluation tools, prompt types (scene-based descriptions and keyword-based descriptions), and AI-generated video paths; (3) Identifying potential themes by categorizing relevant coding; (4) Reviewing and refining themes to ensure internal consistency and distinctiveness; (5) Defining and naming each theme, and (6) Drafting the final analytical report. Descriptive statistics were used to analyze the relative proportion of each dimension based on the topic distribution of the studies in the review. Comparative relationships among qualitative data were presented using percentages and constituent ratios, and all statistical results were visualized in tables and figures.

## 3 Results

A total of 1,206 records were initially identified through database retrieval (PubMed = 243; Google Scholar = 329; Web of Science = 581; China National Knowledge Infrastructure = 53). Of these, 297 were duplicates. Following screening, a further 897 articles were excluded, leaving 12 records sought for retrieval. Of these, two systematic reviews were excluded, leaving 10 articles that met the inclusion criteria and were incorporated into this review (Figure 1).

### 3.1 Characteristics of studies included in the review

Of the 10 studies included in this review, five (5/10) (24–28) were published in 2024, four (4/10) (11, 29–31) in 2025, and one (1/10) (32) in 2026. The studies were conducted across seven countries, with the largest proportion from Asia (6/10) (11, 24, 29–32), followed by Europe and America (3/10) (25, 27, 28), and the smallest proportion from Oceania (1/10) (26). Eight (8/10) of the studies used a mixed-methods design (11, 25–28, 30–32), one (1/10) was quantitative (24), and the remaining study (1/10) was qualitative (29). Three studies (3/10) addressed evaluation of AI-generated videos (26, 29, 31), three (3/10) involved comparisons of different AI platforms (11, 25, 28), four (4/10) explored differences between AI-generated and manually-produced videos (25, 27, 30, 32), and one (1/10) examined comparisons between AI-generated video-based educational materials and traditional materials (24). An overview of the characteristics of the 10 studies is shown in Table 1.

**Table 1:** Characteristics of studies included in the scoping review.

Reference	Year	T2V AI tool/platform; Generated video number	Study design	Study type	Prompts input	Conclusion
Shahrul and Syed Mohamed (11)	2025	InVideo, 1; VEED. IO, 1; VideoGen, 1; Lumen5, 1; Steve. AI, 3	Comparison of videos generated by different AI platforms	Mixed study	Scene-based descriptions	“*AI-generated videos offer poor quality and relevance for orthodontic oral hygiene and dietary advice.*”
Maleki et al. (25)	2024	Pictory, 11	Comparison between the videos generated by AI with the videos on YouTube	Mixed study	Keyword-based descriptions	“*Findings recommend using AI tools for instructional content coverage and structure, with instructor participation in tool selection, content modification, and final evaluation.*”
Mihara (29)	2025	Gemini, LTX Studio, NA	Evaluation of AI generated videos	Mixed study	NA	“*Generative AI, scenario-based learning, and hybrid feedback transform health professions education, enhancing quality and scalability for non-technical skills, enabling multimodal assessments and formative feedback via LLMs, though challenges like ethics and inequities require institutional readiness and customization tools.*”
Dağcı et al. (27)	2025	Sora™ (OpenAI), 5	Evaluation of AI generated videos	Qualitative study	Scene-based descriptions	“*AI-generated videos show potential for nursing education and patient care but face challenges like inaccuracies and deviations from clinical standards, risking patient safety; collaboration between healthcare professionals and AI developers is essential for safe integration.*”
Su et al. (22)	2024	iFLYTEK SPARK, NA	Comparison of verbal education combined with printed materials versus AI-generated personalized patient education videos	Quantitative study	Keyword-based descriptions	“*Personalized AI video education for hyperbaric oxygen patients improves outcomes, reducing anxiety, increasing confidence, and enhancing knowledge, compliance, and satisfaction.*”
Liu et al. (28)	2025	NA, NA	Comparison of health education videos presented by real physicians and AI-generated virtual doctors	Mixed study	NA	“*The image of AI doctors can help patients avoid being influenced by age stereotypes, enabling them to evaluate doctors’ medical expertise more objectively.*”
Macri et al. (24)	2024	NA, NA	Evaluation of patient acceptance of educational videos presented by AI-generated presenters	Mixed study	NA	“*Video-based education may provide information that patients find useful, particularly for physical maneuvers such as face-down positioning. The use of an AI-generated presenter was well-received by the majority of patients.*”
Han et al. (23)	2024	InVideo, 3; ClipTalk, 3; EasyVid, 3	① Comparison of AI-generated and professional patient education videos using different AI video platforms ② Comparison of videos generated by different AI platforms.	Mixed study	Keyword-based descriptions	“*AI text-to-video generators can produce surgical patient education videos with accurate narration and high image clarity but lack medically accurate visuals, requiring improvement for patient use.*”
Taskin and Ak (30)	2026	NA, NA	① Comparison of Anesthesia Education Videos Produced by Humans and Artificial Intelligence ② Exploring the Consistency Between Human Expert Ratings and ChatGPT-5 Evaluations	Mixed study	NA	“*AI-based models like ChatGPT-5 can approximate human expert judgment in evaluating educational content. Although human-made videos are better in source transparency and ethics, AI-generated content is comparable in structure and language. AI-assisted evaluation can be a standardized, efficient tool for screening large medical education video archives.*”
Contreras et al. (26)	2024	HeyGen, 2	Compare video generation capabilities across AI platforms	Mixed study	NA	“*This research indicates that AI-generated videos can be a viable alternative to traditionally produced educational videos. It offers* *an efficient, cost-effective solution for producing educational content. Ethical considerations regarding AI content disclosure should be addressed to maintain transparency.*”

Abbreviation: NA, not applicable; AI, artificial intelligence; T2V, text to video.

### 3.2 Prompt

Five (5/10) (26, 28, 30–32) of the studies did not specify the prompts used for video generation. The remaining five studies (5/10) (11, 24, 25, 27, 29) explicitly documented their prompting strategies, including scene-based descriptions (2/10) (11, 29), and keyword-based descriptions (3/10) (24, 25, 27).

For example, Shahrul and Syed Mohamed used scene-base descriptions in paragraphed prompts such as ‘*Create a video on oral hygiene maintenance and dietary advice during orthodontic treatment for patients, based on resources from the British Orthodontic Society website and patient information leaflets, Public Health England Department of Oral Health Toolkit, American Association of Orthodontists website and YouTube videos, California Association of Orthodontists website, Orthodontics Australia website, Orthodontics New Zealand website, Canadian Association of Orthodontists website, South African Society of Orthodontists website, and Singaporean Association of Orthodontists website*’ (11). Su et al. used keyword-based prompts such as *‘Principles of Hyperbaric Oxygen Therapy’* (24). The five studies (5/10) (11, 24, 25, 29, 31) containing clear prompts all required the AI-generated videos to be based on authoritative evidence and clinical practice guidelines as reference sources.

### 3.3 Generation path and tool selection

**Figure 2:**
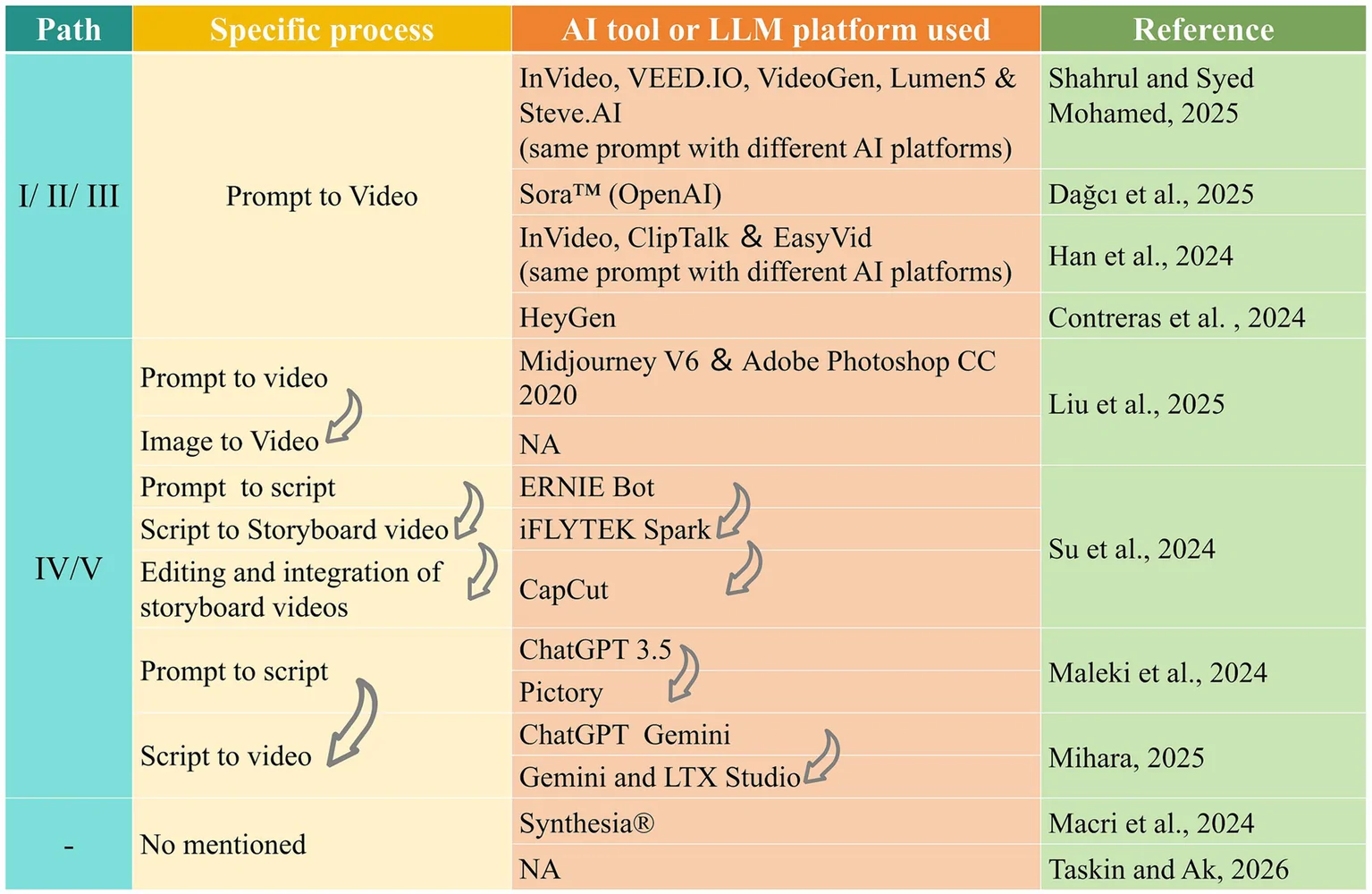
Paths and tools used to generate AI-driven videos in studies covered in this review.

The 10 studies covered all five different generation paths. Four studies covered each of Paths IV/V (24, 27, 30, 31) and Paths I/II/III (11, 25, 28, 29). Two studies (2/10) did not specify a clear generation path (26, 32). The video generation paths of the studies included in this review are summarized in Figure 2.

Overall, 13 T2V AI tools were mentioned for video generation. Four studies (4/10) (24, 27–29) relied on a single T2V generative AI tool. One study each used two (31), three (25) and five (11) T2V AI tools. Two studies (2/10) (26, 30) used AI tools to generate virtual avatars rather than producing videos directly through T2V models. The remaining study did not mention any T2V tools (32). Only two studies (11, 25) used the same AI tool (i.e., InVideo). Details are shown in Table 1 and Figure 2.

### 3.4 Video quality and content evaluation

**Figure 3:**
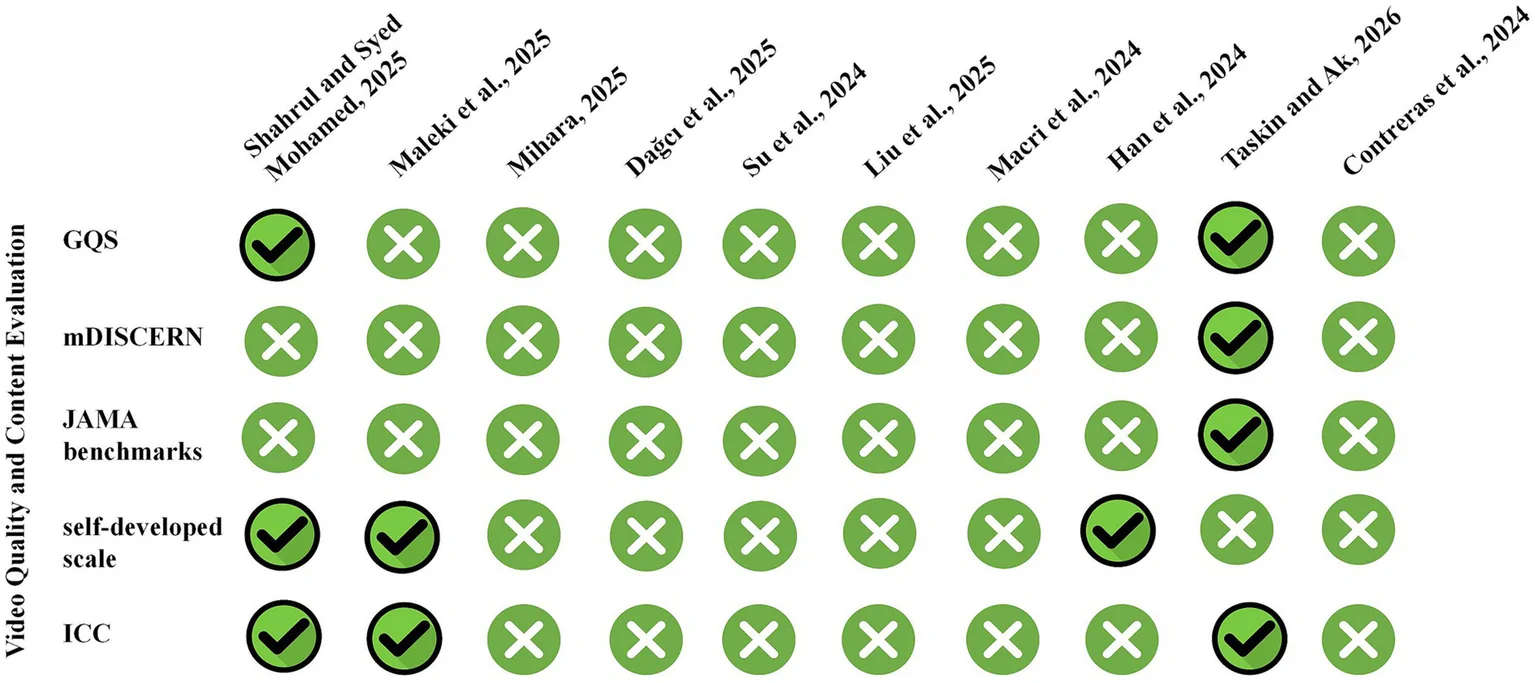
Quality and content evaluation tools for AI-generated videos.

Six studies (6/10) (24, 26, 28–31) did not use any evaluation tools to evaluate the quality or content of AI-generated videos. Two studies (2/10) (11, 32) used the GQS and three (3/10) (11, 25, 27) used self-developed scales. Only one study (1/10) (32) used JAMA benchmarks and mDISCERN for video evaluation. In terms of inter-rater agreement assessment, three studies (3/10) (11, 27, 32) used the ICC method (Figure 3).

## 4 Discussion

To the best of our knowledge, this is among the first scoping reviews to examine AI-generated videos designed for the general public, patient, medical student, or physician. A total of 10 articles were included in the study. The main findings in our review included: (i) The studies were mainly from Asia and focused on patient education; (ii) Half of the studies did not explicitly document their prompts, and those that mentioned prompts primarily presented only scene-based descriptions or keyword-based descriptions; (iii) The AI-generated video used five categories of paths: Open-Source Commercial T2V, Closed-Source Professional T2V, Closed-Source Entry-Level T2V, Stepwise Generation, and Enhanced Generation, and the T2V AI tools used included mainstream options such as Sora and InVideo; (iv) Four articles used evaluation tools (mDISCERN/ GQS/ JAMA benchmarks/ self-developed scales) to assess the videos, and only one of these used three evaluation tools (mDISCERN, GQS and JAMA benchmarks).

### 4.1 Prompt

The prompt is a crucial factor influencing video generation outcomes (33). The prompt provided to generative AI has a significant influence on the resulting output (34). Notably, half of the included studies did not explicitly report the prompts used for video generation. Several factors may contribute to this observation. First, many studies were conducted during the early adoption phase of generative AI, when prompt reporting had not yet emerged as a recognized methodological expectation. Second, some investigators may have regarded prompts as operational details rather than essential components of the research methodology. Third, prompts are often iteratively refined through multiple rounds of interaction with AI systems, making comprehensive documentation challenging.

Nevertheless, inadequate prompt reporting substantially limits reproducibility, transparency, and comparability across studies, given the well-established influence of prompt design on AI-generated outputs. Rodríguez-Donaire recruited 183 students to input different prompts into large language models to generate output to investigate how prompt design influences students’ learning experiences. She found that prompts with robust structural components, including a defined role, an explicit objective and relevant context, produced outputs that students rated as having more depth (35). Hao et al. systematically examined the influence of prompt design on text-to-image generation by comparing original user inputs, manually-engineered prompts, and automatically-optimized prompts. They found that optimized prompts consistently produced images with higher aesthetic scores that were more frequently preferred by humans (36). Five studies examined in this review explicitly stated the prompts used. Three studies used keyword-based descriptive prompts and two used scene-based descriptive prompts (11, 24, 25, 27, 29). Shahrul and colleague employed scene-based descriptive prompts, and the resulting AI-generated videos received low scores for both quality and content (11). This finding suggests that prompt design may play an important role in determining video quality, although this observation is based on a single study and requires further corroboration. Recently, Gao et al. presented RAPO++, a cross-stage prompt optimization framework that unifies training data-aligned refinement and test-time iterative scaling to substantially improve T2V generation without modifying the underlying generative backbone (37). Two other studies have also proposed frameworks and implementation paths for prompt optimization (Sample-Specific Prompt Optimization and Large Language Model Fine-tuning) (37–40). Although prompt engineering is often viewed as a technical process, the educational effectiveness of AI-generated videos is ultimately determined by how well prompts translate pedagogical objectives into audiovisual outputs. Therefore, educational principles and prompt design should not be regarded as separate domains; rather, prompt engineering can be viewed as the operational mechanism through which educational goals are implemented within generative AI systems. Current evidence suggests that selecting appropriate prompt frameworks and devising effective optimization strategies may improve video quality; however, comparative studies remain limited.

### 4.2 Generation path and tool selection

There are many methodologies for generating videos using AI, but the T2V approach is the most prevalent (8). We categorized T2V paths into five distinct types based on user types and demand. Four of the review articles followed paths I/II/III and four to paths IV/V.

Identical paths can be used with diverse tools, and the paths and tools may therefore be combined to fit specific user requirements. Different AI tools all have strengths and weaknesses, and may complement each other in terms of functions. Han et al. (25) compared three T2V tools for creating AI-generated videos (InVideo AI, EasyVid, ClipTalk), and reported that InVideo AI was flexible and supported iterative optimization but suffered from insufficient medical image resources. EasyVid and ClipTalk generated medical-related images but relied on pure AI synthesis with low accuracy. Before using AI tools, users should thoroughly understand their advantages and disadvantages, systematically grasp their functional characteristics, applicable scenarios, and potential limitations, to achieve efficient and rational utilization of the right tools (41, 42). Detailed information about the current mainstream T2V AI tools is summarized in Supplementary Material C.

### 4.3 Video quality and content evaluation

GQS, mDISCERN, and the JAMA benchmarks are widely used for the evaluation of medical science popularization and education videos (43–45). Of the 10 studies included in this review, only one study used all three tools to evaluate AI-generated videos. Each tool focuses on distinct dimensions of video information: GQS evaluates overall video quality (16), mDISCERN assesses information reliability (17), and the JAMA benchmarks gauge information transparency and credibility (18). A multi-tool combined evaluation approach is therefore needed when assessing AI-generated videos. The dimensional complementarity of different tools can effectively offset the limitations of a single evaluation tool. A further three studies developed their own scales to assess the content of AI-generated videos. However, self-developed scales may not objectively and comprehensively cover all the characteristics of AI-generated video content (11, 25). Video quality assessment in the included studies relies on manual scoring tools, which inherently contains subjective judgment and may cause potential assessment errors. To address this limitation, the original studies adopted ICC analysis to quantify intra-rater and inter-rater scoring consistency, thereby mitigating subjective biases and enhancing the credibility of their video evaluation results (46). However, only three studies in this review did so. It is also important to revisit and optimize prompts if evaluation tools give a low score for AI-generated videos (37–40).

The most frequently used evaluation tools such as GQS, DISCERN, and JAMA benchmarks in this review, were originally developed to assess health information quality before the advent of generative AI. Although they remain useful for general quality appraisal, they may not adequately capture AI specific risks, such as hallucinated medical content, visual textual inconsistencies, synthetic imagery fidelity, transparency of AI involvement, and multimodal factual accuracy. Consequently, the current lack of validated assessment tools specifically designed for AI generated medical videos represents a significant methodological gap. Future research should prioritize the development and validation of AI-specific evaluation frameworks capable of assessing both educational effectiveness and risks unique to generative AI systems.

### 4.4 Proposed framework

**Figure 4:**
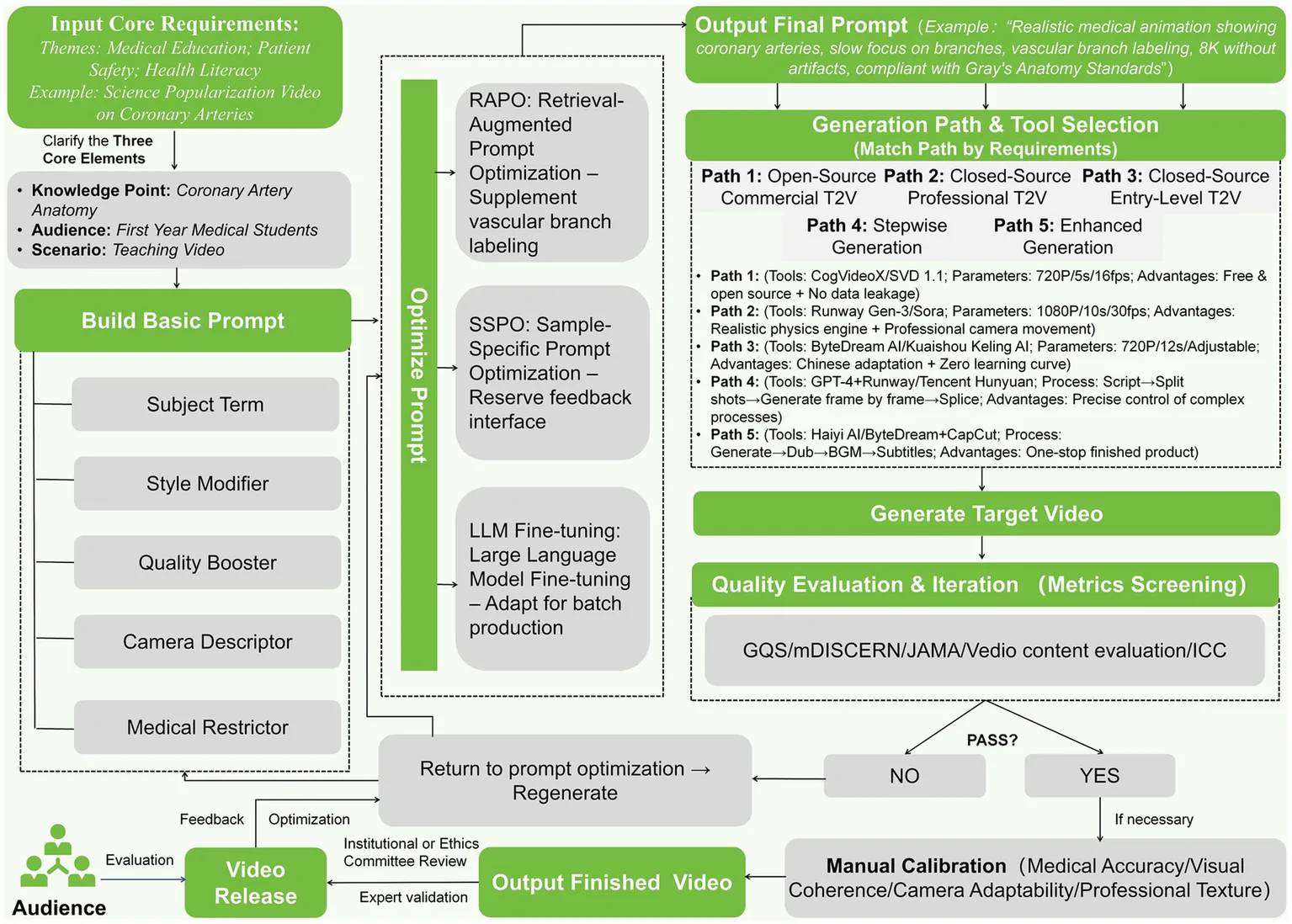
A proposed framework for effective enhancement of AI-generated videos.

Based on the findings of this review and the synthesis of current evidence, we propose a conceptual framework for the generation, evaluation, and implementation of AI-generated medical educational videos (Figure 4). This framework is intended to serve as a practical reference for future research and development rather than a validated workflow. The framework is structured around five interconnected pillars:

- Input specification—Define the target knowledge point, audience, and usage scenario clearly. This ensures that all subsequent steps are grounded in pedagogical clarity.
- Prompt engineering and optimization—Build a structured prompt that includes subject, style, quality, camera, and medical restriction terms. Then refine it iteratively (e.g., using Sample-Specific Prompt Optimization) to improve output precision.
- Generation path and tool selection—Based on the optimized prompt and production needs, select the most suitable approach: open-source models (e.g., CogVideoX), commercial platforms (e.g., Runway, ByteDream), or stepwise pipelines (e.g., GPT-4 plus frame-by-frame generation and splicing). Optionally, fine-tune large language models for batch production efficiency.
- Quality assurance—Apply a multi-dimensional battery of standardized instruments (GQS, mDISCERN, JAMA benchmarks, custom scales, and ICC for inter-rater reliability). Also conduct expert manual calibration to check medical accuracy, visual coherence, camera adaptability, and professional texture.
- Feedback and validation—Obtain institutional or ethics committee review and collect audience feedback. This ensures that the final video meets both ethical standards and educational effectiveness (47, 48).

The stages within this framework are closely interconnected, and comprehensive evaluation of each component is essential to ensure that AI-generated medical videos achieve appropriate quality standards. When generating AI medical videos, we should check factual accuracy, visual clarity, anatomical correctness, risk communication, explanation clarity, and audience suitability. Future research should further develop standardized evaluation frameworks that extend beyond technical and content quality assessments to incorporate learner- and patient-centered educational outcomes, thereby enabling a more comprehensive understanding of the educational and clinical value of AI-generated medical videos.

### 4.5 Regulatory and ethical considerations

The use of generative AI in producing educational videos raises critical ethical and regulatory challenges, encompassing transparency, copyright, misinformation, privacy, and governance. Disclosure of AI involvement and adherence to copyright regulations are currently inconsistent across medical education settings (49). Furthermore, content generated by AI frequently contains clinically implausible errors, such as suggesting a pregnancy test for a male patient, highlighting the absolute necessity for expert validation (13, 50). Regarding privacy, identifiable patient data must never be processed through unprotected commercial tools, and any data used for training must comply with Health Insurance Portability and Accountability Act or Genera Data Protection Regulation standards (49). In terms of institutional oversight, clear frameworks are required to assign accountability for the disseminated medical information (51). Looking forward, research must move beyond technical comparisons of generative platforms and focus on clinically meaningful educational outcomes, including patient comprehension, treatment adherence, shared decision making, and health literacy. Ultimately, evaluating how these videos impact actual user outcomes will determine their true value in modern clinical practice.

### 4.6 Limitations

Several limitations of this scoping review must be acknowledged, primarily stemming from the nascent nature of the current evidence base. Firstly, considerable methodological heterogeneity and variability in study quality were observed across the included studies, particularly regarding study design, target populations, and evaluation methods. Furthermore, prompt formulations were insufficiently reported, and the transparency of proprietary AI tools remained limited. Future researches should employ more standardized methodologies, improve reporting practices, incorporate population specific evaluation approaches, and conduct larger multicenter studies. Secondly, our search strategy was restricted to English and Chinese publications, which may lead to language bias. Although unpublished materials and preprints were excluded to maintain evidence quality, this approach may have resulted in the omission of the most recent developments in this rapidly evolving field. Thirdly, given the rapid pace of advancement in generative AI, the tools and generation approaches evaluated in the included studies may quickly become outdated. Therefore, ongoing evidence updates and future reviews are needed to ensure that conclusions remain current and relevant. Fourthly, our review concentrated on optimizing the generation process of AI-generated medical educational videos, including prompt design, generation tools, framework, and quality assessment approaches. These provide an important foundation for improving the reliability, consistency, and standardization of AI-generated educational content. However, evidence regarding educational and clinical outcomes remains limited. Future studies should build upon these technical advances by examining their impact on knowledge acquisition, patient understanding, health literacy, behavioral change, patient satisfaction, and other clinically meaningful outcomes.

## 5 Conclusion

This scoping review is the first of which we are aware to evaluate AI-generated videos for medical science popularization and education. Based on our findings, we propose a procedural framework that offers concrete implications for key stakeholders: guiding researchers in rigorous tool validation, and assisting public health educators and healthcare institutions in disseminating safe, high-quality content. To advance this field, future work must prioritize transparent prompt reporting, explicit documentation of model versions, and rigorous expert review of generated videos. Furthermore, establishing standardized evaluation batteries and clear procedures for identifying medical inaccuracies is essential. While our framework provides a foundation for more transparent AI-generated medical educational videos, its feasibility and effectiveness require prospective evaluation.

## Data Availability

The original contributions presented in the study are included in the article/Supplementary material, further inquiries can be directed to the corresponding author.
